# Can Reflex UroVysion fluorescence *in situ* hybridization predict tumor recurrence during follow-up?

**Published:** 2007

**Authors:** Annamma Kurien, Joseph Thomas

**Affiliations:** Department of Pathology, Melaka Manipal Medical College, Manipal, India; * Department of Urology, Kasturba Medical College, Manipal, India. E-mail: drannammakurien@yahoo.co.in

## SUMMARY

The primary aim of this study was to demonstrate the usefulness of multitarget fluorescence *in situ* hybridization (FISH UroVysion) testing in the early detection of recurrent urothelial carcinoma (UC). The study population comprised patients with bladder tumor on follow-up, who had negative or atypical urine cytology with no evidence of recurrence by cystoscopy. Some of these patients had positive UroVysion FISH in the urine cell sample. The paper looks at the course of these “anticipatory positive” cases with special reference to the recurrence and the time to tumor recurrence. Fifty-six (26.5%) of the 211 patients with negative cystoscopy and negative or atypical cytology had positive FISH results. This group formed the anticipatory positive subset of patients. Recurrent urothelial carcinoma developed in 35 (62.5%) of these 56 patients. Of these recurrent tumors 22 were high-grade UC and 12 low-grade UC. In about 65% of this anticipatory positive group, recurrent bladder UC bladder developed within 29 months. The recurrence rate was 48% in six months and 54% within 10 months of the positive FISH result. In contrast recurrent UC developed in only eight (5.2%) of the 155 cystoscopically negative, cytologically negative or atypical and FISH negative cases.

## COMMENTS

Urothelial carcinoma has a high rate of recurrence and progression, necessitating frequent patient surveillance by follow-up is by cystoscopy and urine cytology.[1] The invasive nature of cystoscopy and the relative low sensitivity of cytology are the limitations of this approach. This has led to many adjunctive assays to stratify individuals into high- and low-risk categories.[2] UroVysion is a multitarget FISH assay to detect chromosomal alterations by three DNA probes directed towards the pericentromeric regions of Chromosome 3,7 and 17 and a fourth probe to 9p21 locus. This molecular cytology by the combination of probes allows detection of chromosomal aneusomy and or deletion of 9p21.[3] Though FISH analysis is currently the most sensitive marker for bladder tumors, the cost of the DNA probes and the laboratory equipment required, limits its use in the everyday routine.[4] However, it is useful for the early identification of patients who are likely to progress. It also helps to lengthen the surveillance period in those with low-risk disease.[5] There is no improvement of FISH over cytology in the cytology-positive cases for detection of recurrence during follow-up.[6] It is worth noting here that many low-grade lesions do not shed cells and do not exhibit chromosomal changes detected by FISH testing. The data presented in the study shows that reflex UroVysion on urine specimens helps to identify the anticipatory positive case so that more aggressive therapeutic options can be planned.

## References

[1] Canfield SE, Dinney CP, Droller MJ (2003). Surveillance and management of recurrence for upper tract transitional cell carcinoma. Urol Clin North Am.

[2] Black PC, Brown GA, Dinney CP (2006). Molecular markers of urothelial cancer and their use in the monitoring of superficial urothelial cancer. J Clin Oncol.

[3] Bollmann M, Heller H, Bajnkfalvi A, Griefingholt H, Bollmann R (2005). Quantitative molecular urinary cytology by fluorescence in situ hybridization: A tool for tailoring surveillance of patients with superficial bladder cancer?. BJU Int.

[4] Krause FS, Rauch A, Schrott KM, Engehausen DG (2006). Clinical decisions for treatment of different staged bladder cancer based on multi target fluorescence in situ hybridization assays?. World J Urol.

[5] Sarosdy MF, Kahn PR, Ziffer MD, Love WR, Barkin J, Abara EO (2006). Use of a multi target fluorescence in situ hybridization assay to diagnose bladder cancer in patients with hematuria. J Urol.

[6] Moonen PM, Merkx GF, Peelen P, Karthaus HF, Smeets DF, Witjes JA (2007). UroVysion compared with cytology and quantitative cytology in the surveillance of non-muscle-invasive bladder cancer. Eur Urol.

